# Incorporation of Ion Transport Chains into Multivariate MOF for Improved Water Oxidation

**DOI:** 10.1021/acsmaterialslett.5c01586

**Published:** 2026-02-16

**Authors:** Benjamin Thomas, Sumanta Basak, Amanda J. Morris

**Affiliations:** † Department of Chemistry, 1757 Virginia Polytechnic Institute and State University, Blacksburg, Virginia 24061, United States; ‡ Macromolecules Innovation Institute, 1757 Virginia Polytechnic Institute and State University, Blacksburg, Virginia 24061, United States

## Abstract

The climate crisis demands clean energy technologies to cut CO_2_ emissions from fossil fuels. Hydrogen fuel cells and solar-driven CO_2_ reduction are promising, but both rely on efficient water oxidation. Polypyridyl ruthenium complexes are active catalysts for water oxidation; however, they exhibit poor stability and recyclability. Our group improved performance by embedding these complexes into metal–organic frameworks (MOFs). As water oxidation is pH-dependent, proton management further enhances reactivity. To address the issue, we introduced proton transfer pathways into the MOF structure. Specifically, we incorporated −SO_3_H groups onto the biphenyl linkers of UiO-67 loaded with [Ru­(tpy)­(dcbpy)­OH_2_]­PF_6_ catalyst (where tpy = 2,2′:6′,2″-terpyridine; dcbpy = 5,5-dicarboxy-2,2′-bipyridine). The sulfonated MOF exhibited a 2.5-fold increase in oxygen evolution compared to the nonsulfonated analogue. After 1 h of electrolysis, the sulfonated MOF exhibited a turnover number of 25 for oxygen evolution reaction compared to 10 for the native MOF, demonstrating the benefits of built-in proton management.

## Graphical abstract

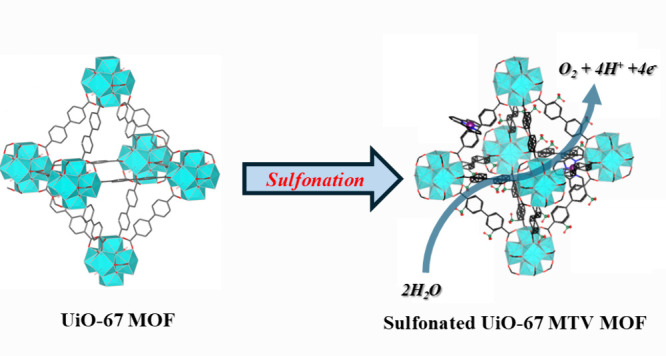

Water oxidation plays a crucial role in the future of clean energy. The process, shown in eq 1, involves the conversion of water molecules into oxygen gas, protons, and electrons. The electrons generated can be used in reduction processes such as carbon dioxide (CO_2_) reduction and proton reduction for use in combustion reactions and fuel cells.^1–3^ Highly efficient water oxidation replaces the need for sacrificial electron donors in the reduction reactions, making the processes genuinely catalytic.

1 $$\begin{array}{l} 2H_{2}O\to O_{2}+4H^{+}+4e^{-} \end{array}$$

Ruthenium polypyridyl complexes are well-studied water oxidation catalysts that exhibit some of the fastest turnover rates within the literature.^4,5^ These catalysts are hindered by their instability in solution, the high cost of Ru, and difficult recyclability that stems from the need to separate a homogeneous compound from the solution. To address these issues, monolayers of molecular catalysts have been built on electrode surfaces.^6–8^ These monolayers showed similar redox properties to their solution counterparts while eliminating the diffusional aspect of the charge transfer steps. However, the approach is constrained by the electrode’s surface area, which caps the amount of immobilized catalyst and limits overall catalytic performance.

Metal–organic frameworks (MOFs) are highly crystalline, porous materials with high surface area. These properties have led to MOF investigations for several applications, including gas storage and separation,^9^ drug delivery,^9^ electrode materials,^10^ and catalysis.^11–14^ The highly ordered nature of these materials leads to multiple isolated, accessible active sites spaced throughout the framework. Incorporating commonly used homogeneous catalysts into the backbone of the MOF leads to higher turnovers than their natural counterparts because their stability is increased through site isolation.^15–18^ Several MOF films have been studied for their activity in CO_2_ reduction and have shown significant improvements over their homogeneous counterparts.^19^ Fewer studies have been done on MOF materials for water oxidation.^20,21^ Our group has previously incorporated RuTPY, [Ru­(tpy)­(dcbpy)­OH_2_]­PF_6_ catalyst (where tpy = 2,2′:6′,2″-terpyridine; dcbpy = 5,5-dicarboxy-2,2′-bipyridine), a highly active water oxidation catalyst, into the backbone of UiO-67, a biphenyl-based Zr-MOF.^22,23^ The results showed improved water oxidation and the ability to recycle the material for multiple catalytic runs.^23^

Proton transport is critical for efficient water oxidation, as proton buildup around the active site increases the thermodynamic barrier for oxidation. In Photosystem II, responsible for the photochemical water oxidation step in photosynthesis, the Mn_4_CaO_5_ active site generates oxygen while releasing protons that are shuttled away to create a proton gradient necessary for ATP production.^11^ The proton transport chain in Photosystem II consists of surrounding amino acids that facilitate rapid proton transfer. Charged residues such as aspartic acid, glutamic acid, lysine, and arginine form the bulk of this transport channel, with their arrangement creating a path of water molecules that enables efficient proton movement. A lack of defined surroundings beyond the solvent presents a challenge for improving the reactivity of traditionally synthesized molecular catalysts for water oxidation. Acting as a protein-like backbone, the MOF framework can be functionalized with groups that enable directed ion transport. The modular nature of MOFs allows for the incorporation of both active water oxidation catalysts and molecules which can shuttle protons away from the active sites. A multivariate synthetic approach can integrate linkers bearing catalytic centers with linkers containing functional groups for efficient proton transport.

Herein, we report the synthesis of a new multivariate MOF, RuTPY-UiO-67-SO_3_H. The MOF contains two linkers; the known water oxidation catalyst, RuTPY, [Ru­(tpy)­(dcbpy)­Cl]­PF_6_, where tpy = 2,2′:6′,2″-terpyridine and dcbpy = 5,5-dicarboxy-2,2′ - bipyridine, and a disulfonated biphenyl dicarboxylic acid, 3,3′-disulfo-[1,1’-biphenyl]-4,4’-dicarboxylic acid. Incorporating both linkers primes the structure for synergistic effects by having the sulfonate groups proximal to the active RuTPY catalyst. The deprotonated sulfonate groups bound to the native biphenyl linkers of UiO-67 create an ion transport chain away from active catalysis sites and thereby enhancing oxygen evolution.

RuTPY-UiO-67 was prepared following a reported procedure, and the sulfonated variant, RuTPY-UiO-67-SO_3_H, was synthesized analogously by substituting biphenyl dicarboxylic acid with 3,3′-disulfo-[1,1′-biphenyl]-4,4′-dicarboxylic acid (Scheme 1, Figure S1). Full synthetic details are provided in the Supporting Information (Figure S2).

**Scheme 1:**
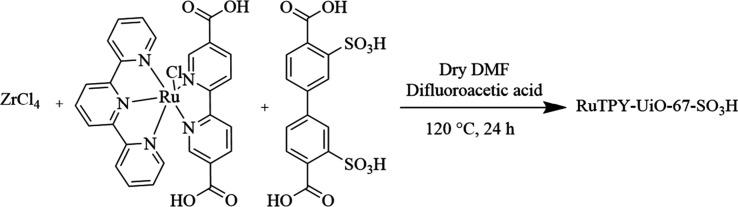
Direct Solvothermal Synthesis of the Multivariate MOF RuTPY-UiO-67-SO_3_H

The powder X-ray diffraction (PXRD) patterns of the synthesized MOF thin films agree with the simulated pattern of parent UiO-67, indicating the ruthenium catalyst and sulfonated biphenyl linker were incorporated into the framework and did not alter the crystal structure of the three-dimensional framework (Figure 1a). Scanning electron microscope (SEM) images of the thin films show the octahedral crystals indicative of a UiO-type MOF. Some of the crystals appear to be intergrown among each other, but the general octahedral structure holds for most of the microcrystals (Figure 1b,c). The relative stoichiometry between the RuTPY units and the sulfonated biphenyl linkers was determined by digestion ^1^H NMR spectroscopy, a widely accepted technique for quantifying linker and catalyst incorporation in mixed-linker MOFs.^24^ Integration of the characteristic proton resonances corresponding to the RuTPY complex and the sulfonated biphenyl linker indicates that RuTPY is incorporated at an approximate 1:5 ratio relative to the sulfonated biphenyl linkers. This corresponds to an average loading of one RuTPY unit per Zr_6_ node, consistent with the designed framework composition and confirming successful incorporation of the RuTPY catalyst and sulfonated linker within the MOF structure. X-ray photoelectron spectroscopy (XPS) was performed to confirm the presence and chemical state of the sulfonated linkers. The spectrum shows a broad feature in the 167–170 eV region, corresponding to the expected doublet of sulfonate (−SO_3_H/–SO_3_^–^) groups, consistent with reported values for sulfonated MOFs (Figure S3).^25^ This qualitatively confirms the presence of the sulfonated linker, complementing the quantitative stoichiometry obtained from digestion ^1^H NMR. The bulkiness of the terpyridine group limits the amount of catalyst that can be incorporated due to the constricted pore size of UiO-67. The total ruthenium on the electrode was determined by UV–vis of the degraded sample, Figure S4. The average amount of ruthenium found on the RuTPY-UiO-67 was (5.0 ± 1.1) × 10^–8^ mol/cm^2^ RuTPY. The average amount of ruthenium found on the RuTPY-UiO-67-SO_3_H is (3.3 ± 0.8) × 10^–8^ mol/cm^2^ RuTPY.

**Figure 1:**
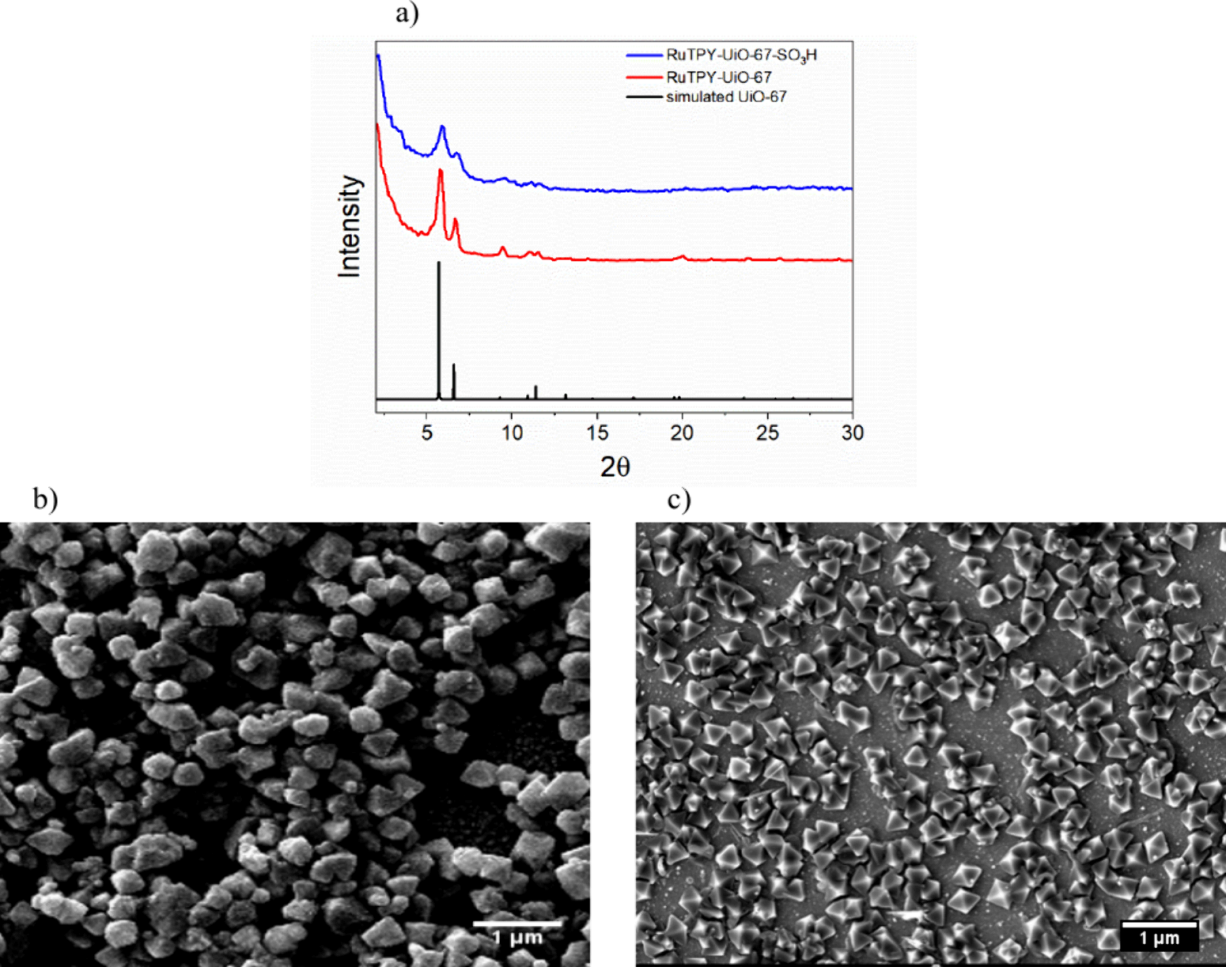
(a) PXRD patterns for the synthesized MOF thin films as compared to simulated UiO-67 (CCDC 1021002) and SEM images of (b) RuTPY-UiO-67 and (c) RuTPY-UiO-67-SO_3_H thin films.

Scan rate-dependent cyclic voltammograms in aqueous 0.1 M LiClO_4_ are shown in Figure 2, displaying the redox responses for RuTPY-UiO-67 and RuTPY-UiO-67-SO_3_H. Both materials exhibit the characteristic Ru^II/III^ redox couple, appearing at *E*_1/2_ = 0.766 V vs NHE for RuTPY-UiO-67 and *E*_1/2_ = 0.734 V vs NHE for RuTPY-UiO-67-SO_3_H. Differential Pulse Voltammetry (DPV) in Figure S5 shows a shoulder peak around 1.2 V vs NHE, which is attributed to the Ru^III/IV^ couple.

**Figure 2:**
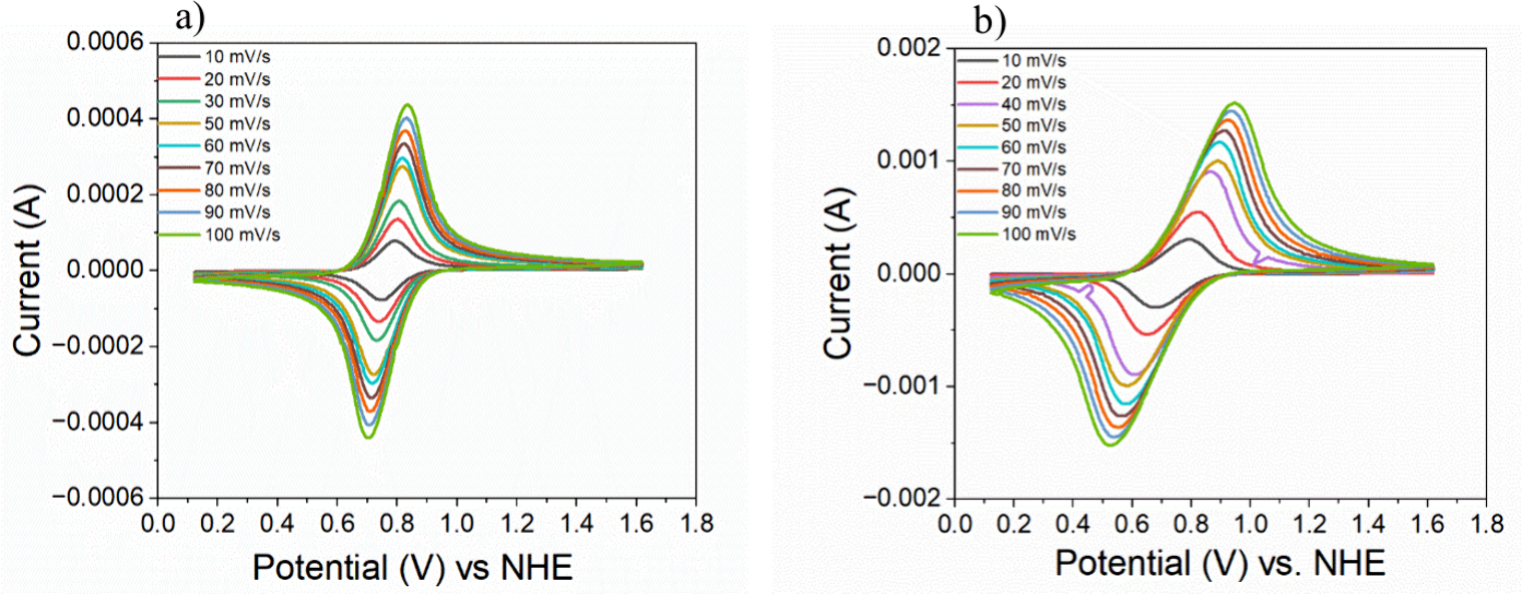
Scan rate dependent cyclic voltammograms for (a) RuTPY-UiO-67 and (b) RuTPY-UiO-67-SO_3_H.

Due to the insulating nature of the Zr nodes in UiO-67 and the isolated positions of the RuTPY redox centers, charge transfer in RuTPY-UiO-67 type MOFs occurs through a redox hopping mechanism.^26,27^ The charge hops from redox center to redox center coupled with the diffusion of a charge balancing ion. For RuTPY-UiO-67 (Figure 2a), the redox couple appears at approximately 0.766 V vs NHE with a small peak-to-peak separation, indicating a largely reversible, surface-confined electron-transfer process. The peak currents scale linearly with scan rate, confirming that the redox centers are immobilized within the MOF film. In contrast, the sulfonated analogue, RuTPY-UiO-67-SO_3_H (Figure 2b), exhibited broader peaks with greater separation that increase with scan rate, characteristic of a quasi-reversible process. The behavior suggests that electron transfer in the sulfonated MOF is influenced by coupled ion diffusion through the framework. Scan rate-dependent cyclic voltammetry can be used to determine the rate of the redox hopping within the framework known as the apparent diffusion coefficient or *D*_*app*_. At low scan rates (*v*), the current response is governed by surface-controlled charge transfer, where ion or electron diffusion occurs rapidly enough to match the applied potential sweep, resulting in a peak current that varies linearly with *v*. As *v* increases, the system transitions to a diffusion-controlled regime because the *D*_*app*_ becomes insufficient to follow the rapid potential changes, and the current instead scales linearly with *v* ^*1/2*^. The scan rate at which this transition occurs can be used to estimate the *D*_*app*_. The RuTPY-UiO-67-SO_3_H showed a shift in the current response from surface to diffusion within the 50–60 mV/s range (Figure S6). Using eq 2, the transition scan rate can be used to find a *D*_*app*_ value by setting λ to 1 and gave a *D*_*app*_ of 8.3 × 10^–8^ cm^2^/s:

2 $$\lambda =d_{f}\sqrt{\frac{Fv}{D_{app}RT}}$$

where λ_e_ is a dimensionless parameter and can be defined to relate the film thickness to the thickness of the electron diffusion layer and R and T being the universal gas constant and absolute temperature.

As per previous reports, the diffusion of charge throughout the MOF film can be calculated using spectroelectrochemistry.^13–15^ RuTPY is an electrochromic molecule where, upon oxidation of Ru^II^ to Ru^III^, spectral changes consistent with a visible color change from deep purple to pale yellow are observed.^16^ The absorbance spectra in Figure S7a show the absorbance of the RuTPY molecule in solution in the Ru^II^ and Ru^III^ oxidation states. The characteristic MLCT band has a λ_max_ of 527 nm. The oxidation of the Ru^II^ complex results in a decrease of approximately 85% in the extinction coefficient. Using the modified Cottrell equation (eq 3), the change in absorbance upon oxidation at a potential beyond the Ru^II/III^ redox couple can be used to calculate *D*_*app*_.

3 $$\Delta A=\frac{2A_{\max}}{d_{f}}\sqrt{\frac{D_{app}t}{\pi }}$$

The parent and sulfonated MOF films were subjected to oxidation in 0.1 M LiClO_4_ in acetonitrile at 1.2 V vs Ag/Ag^+^, which is beyond the oxidative wave for Ru^II/III^. The change in absorption was measured at 527 nm. The oxidized films were then reduced back to the ground state Ru^II^ by applying the open circuit potential. The RuTPY-UiO-67-SO_3_H film exhibited an immediate decrease in absorbance of approximately 85%, consistent with complete oxidation of Ru^II^ to Ru^III^, as the Ru^III^ species exhibits a molar absorptivity about 85% lower than that of Ru^II^. The absorbance fully recovered to its initial value upon reduction, confirming that the MOF framework remained intact and that the observed spectral change arises solely from the reversible oxidation/reduction of RuTPY to the Ru^III^ state (Figure S7b). The complete and reversible absorbance modulation indicates that essentially all RuTPY sites within the film are electrochemically accessible and active. Consecutive oxidation–reduction cycles demonstrated consistent absorbance behavior over at least five cycles, indicating excellent electrochemical and structural stability. The *D*_*app*_ for the RuTPY-UiO-67-SO_3_H was 3.1(±0.5) × 10^–7^ cm^2^/s.^19^ In contrast, RuTPY-UiO-67 exhibited only a partial color change, corresponding to approximately 50% oxidation of the Ru centers. The calculated diffusion coefficient for the RuTPY-UiO-67 was 4.7(±0.9) × 10^–11^ cm^2^/s.

Controlled-potential electrolysis (CPE) was carried out to evaluate the electrocatalytic water oxidation activity of the MOF thin films. When a potential of 1.71 V versus NHE (corresponding to an overpotential of 880 mV) was applied, the RuTPY-UiO-67-SO_3_H thin film exhibited a substantially higher catalytic current density compared to both bare FTO and nonsulfonated MOF thin film under identical conditions (Figure 3a). The concentration of oxygen over time for both the RuTPY-UiO-67 and RuTPY-UiO-67-SO_3_H is shown in Figure 3b. The turnover for the RuTPY-UiO-67-SO_3_H reaches an average of (25 ± 3) mol of O_2_ per Ru mol in an hour, two times that of the native RuTPY-UiO-67, which only reaches (10 ± 2) turnovers for oxygen over the same period. The RuTPY-UiO-67-SO_3_H film exhibited a Faradaic efficiency for O_2_ evolution of (73.2 ± 3.2)%. Although the total Ru loadings are similar within experimental uncertainty, catalytic performance is evaluated using turnover numbers normalized to electrochemically active Ru, ensuring that differences in oxygen evolution activity are intrinsic rather than arising from catalyst loading. The water oxidation catalysis by Ru­(tpy)­(dcbpy)-type complexes is widely described in terms of sequential proton-coupled electron transfer (PCET) steps. Upon stepwise oxidation of the Ru^II^ center to Ru^III^ and Ru^IV^ states, a high-valent Ru^IV^=O intermediate is generated, which then undergoes O–O bond formation via nucleophilic attack by a water molecule. The mode of action for Ru-based polypyridyl complexes has been confirmed through kinetic studies, electrochemistry, and spectroscopic techniques applied to either free Ru polypyridyl catalysts and their immobilized versions.^28,29^

**Figure 3:**
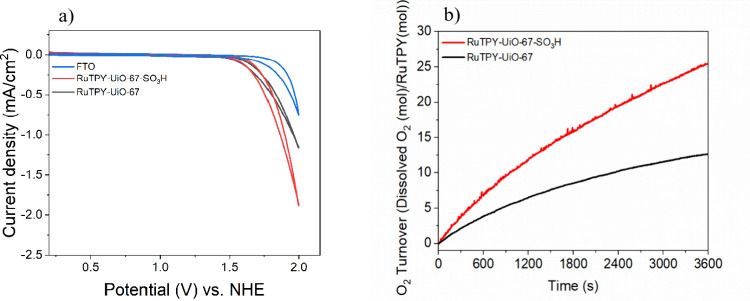
(a) Cyclic voltammograms of FTO and MOF samples in 0.1 M LiClO_4_ aqueous solution and (b) turnover numbers for oxygen generation during bulk electrolysis of MOF films.

The thin films were tested for repeated reactivity and showed similar oxidation rates for multiple electrolysis periods when a fresh electrolyte solution was used (Figure 4a). The sustained reactivity confirms that the MOF remains structurally intact in solution and that the −SO_3_H functionalities continue to enhance catalytic performance (Figure S8).

**Figure 4:**
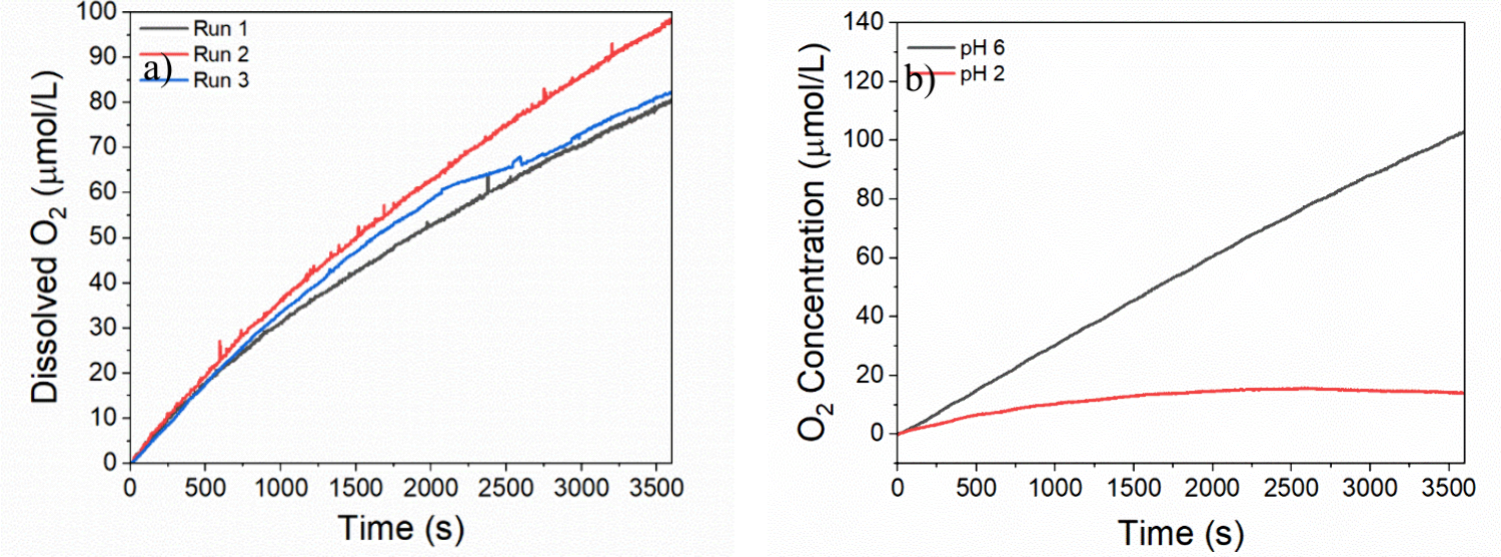
(a) RuTPY-UiO-67-SO_3_H film reused for a third time with no loss of catalytic activity and (b) oxygen evolution reaction of RuTPY-UiO-67-SO_3_H at pH 2 (red) and pH 6 (black).

To determine the role of sulfonic acid groups in increased reactivity, one must consider both electrochemical surface coverage and proton transport. Absorbance data show that RuTPY-UiO-67-SO_3_H MOF exhibits full electrochemical accessibility of its Ru centers compared to the nonsulfonated analog, with ∼ 55% accessible sites. The data translated to 1.1 × 10^–7^ mol of active Ru, compared to 1.3 × 10^–7^ mol for the sulfonated analogue. The increase in catalyst concentration alone is too small to account for the ∼2.5-fold higher O_2_ production rate. The mismatch indicates that the −SO_3_H groups contribute directly to the improved water oxidation kinetics. Indeed, a pronounced kinetic isotope effect (KIE = 4.213 ± 0.220) was observed when catalytic currents were compared in H_2_O and D_2_O electrolytes under identical conditions (Figure S9).^30,31^ The substantial isotope effect strongly indicates that O–H bond cleavage and associated proton transfer are directly involved in the rate-determining step of water oxidation.^32^ The introduction of −SO_3_H groups into the catalyst framework likely contributes to the enhanced activity by facilitating PCET through well-organized hydrogen-bonding networks and proton-relay pathways. Comparable H/D isotope effects have been widely reported for molecular and heterogeneous water oxidation catalysts and are consistently interpreted as signatures of proton-limited rate-determining steps.^33^ Furthermore, tailoring the catalyst environment with acidic functional groups such as −SO_3_H is known to lower the barrier for PCET by stabilizing proton transfer pathways, thereby accelerating water oxidation kinetics. Other reports have indicated that the dangling −SO_3_H groups have shown increased proton transport through a porous film.^34,35^ In contrast, the MOF with no sulfonate showed a KIE of (1.1 ± 0.09), which indicates very little involvement of proton transfer during the rate-determining step. To further confirm the involvement of the sulfonate groups in catalysis, the pH of the solution was lowered to shut down the ion transport capabilities by protonating the groups thus removing the negative charge of oxygen atoms. A drastic lowering in reactivity was seen when the pH was lowered to 2, with almost no oxygen being produced. The pH was then returned to pH 6, and the initial high reactivity was restored (Figure 4b). It has been demonstrated through several studies that incorporating acidic functional groups, such as sulfonate, into MOFs affects the movement of protons through or around the framework, as well as the local reaction conditions in electrocatalysis systems. For example, sulfonate-functionalized MOF-808 coatings were used to enrich protons near a copper electrocatalyst, improving selectivity for electrochemical nitrate reduction by increasing local proton availability.^36^ The incorporation of propyl-sulfonic acid groups into MIL-101­(Cr) after synthesis resulted in a significant increase in proton conductivity when in humid conditions, demonstrating the ability of acidic groups to effectively facilitate the transport of protons.^37^ Likewise, using acidic ionic liquids with MOFs has demonstrated a marked improvement in proton transport, showing that sulfonates can be used as proton relays within pores to improve performance significantly.^38^ These works underscore the utility of sulfonate and related functional groups in tuning proton dynamics within MOFs, consistent with our focus on proton management to enhance water oxidation catalysis.

The MOF films were also confirmed to retain their crystallinity as shown in Figure S10a with the PXRD of the MOF thin films retaining its peak positions and intensities after 2 h of continuous electrolysis. XPS measurements were carried out on the films both prior to and following catalysis. The Ru 3p_3/2_ peak was observed at 462.39 eV before catalysis and at 462.56 eV afterward, showing that the ruthenium sites retained their chemical stability under the applied electrochemical conditions. Such negligible peak shifts indicate that the Ru centers did not experience notable alterations in oxidation state or coordination during the catalytic process (Figure S10b). To evaluate possible catalyst leaching, inductively coupled plasma mass spectrometry (ICP-MS) analysis was performed and showed that only ∼ 4.48% of the total Ru content was released into solution. Collectively, these results demonstrate that most Ru centers remained incorporated within the MOF lattice during catalysis. Overall, the data confirm that the MOF framework maintains both structural integrity and chemical resilience under electrocatalytic water oxidation conditions at the working pH.

We report the incorporation of sulfonate groups into the backbone of UiO-67 loaded with a known water oxidation catalyst, RuTPY by decorating the native biphenyl linkers within the framework. The incorporation of the proximal sulfonate groups within the confined space of the MOF intends to create a proton shuttle to remove generated H^+^ near the active site to increase turnover, similar to proximal amino acids within Photosystem II. The RuTPY-UiO-67-SO_3_H showed higher oxygen production per electrochemically active Ru atom compared to the RuTPY-UiO-67, with the −SO_3_H film reaching 25 turnovers over an hour, while the native RuTPY-UiO-67 only reached close to 10. The sulfonate groups lead to higher ion diffusion throughout the framework itself, allowing all RuTPY centers to be electrochemically active for oxidation while the RuTPY-UiO-67 showed only 55% of the RuTPY could be electrochemically accessed. The work shows the power of a multivariate approach to MOFs and the synergistic effect that can be seen with the sulfonate functional groups and the water oxidation catalyst. The confined nature of MOFs should further be exploited to improve catalytic activity.

## Supplementary Material



## Notes

Experimental methods and details, synthesis of catalyst and linkers, absorbance spectra, DPV curve, and chronoamperometry plots (PDF)
