# Supplementary material for: Incorporation of Ion Transport Chains into Multivariate MOF for Improved Water Oxidation
Source: ACS Mater Lett. 2026 Feb 16;8(3):896–902. doi: 10.1021/acsmaterialslett.5c01586 (PMC12958330; doi:10.1021/acsmaterialslett.5c01586)
Supplement: Supplementary file 1 [file tz5c01586_si_001.pdf]

Supporting Information

for

Incorporation of Ion Transport Chains into Multivariate MOF for Improved Water Oxidation

Benjamin Thomas [a], Sumanta Basak [a], and Amanda J. Morris [a,b] *

[a]Department of Chemistry, Virginia Polytechnic Institute and State University, Blacksburg, Virginia 24061, United States

[b]Macromolecules Innovation Institute, Virginia Polytechnic Institute and State University, Blacksburg, Virginia 24061, United States

*Email: ajmorris@vt.edu

1. Characterization Methods

1.1 Powder X-ray Diffraction (PXRD)

PXRD patterns were collected on a Rigaku Miniflex instrument (Cu Kα, λ= 1.5418 Å) from 2° to 30° with a resolution of 0.05° at a rate of 0.5° per min.

1.2 Electrochemical Measurements

Electrochemical measurements were conducted using a Pine Instruments Wavenow potentiostat with a three-electrode arrangement, where the MOF film was coated on an FTO slide as the working electrode, an Ag/AgCl reference electrode, and a platinum mesh counter electrode. Water oxidation studies were done in 0.1 M $LiClO_4$ adjusted to pH 6 using HCl. Bulk electrolysis was conducted at 1.70 V vs. NHE. O_2 production was measured with a Clarke-type dissolved oxygen probe (Unisense OX-NP). A blank FTO slide in a solution of potassium ferricyanide ($E_{1/2}$ = 0.361 V vs. NHE) was tested before every oxidation run to calibrate the reference electrode.

1.3 Absorbance Measurements

Absorbance measurements were taken on a Cary UV-Vis-NIR spectrophotometer. The full absorption spectra were taken from 800 nm to 200 nm at a speed of 600 nm per minute. The molar extinction coefficients were collected in a specialized quartz cuvette from Pine Research Instrumentation. A solution of 9.54×10^{-5} M RuTPY in acetonitrile was used to fill the cell. A honeycomb electrode from Pine Research Instrumentation with a platinum working electrode and counter electrode was placed in the cell along with an Ag/AgCl reference electrode. The cuvette was loaded into the Uv-Vis and positioned so the light beam would go through the honeycomb electrode. An absorbance spectrum was taken to get the standard spectra of the RuTPY molecule. A potential of 1.2 V vs. Ag/AgCl was applied for 5 min to oxidize the RuTPY at the electrode

surface. The absorbance spectrum of the cell was then taken while holding the potential at 1.2 V vs. Ag/AgCl.

For kinetics measurements, the absorbance at the selected wavelength was tracked with data points collected every 0.033 seconds. The MOF film was placed in a glass cuvette so the beamline would travel directly through the film. The cuvette was filled with 0.1 M $LiClO_4$ MeCN, and an Ag/Ag+ reference electrode and a platinum wire counter electrode were placed in the cell without blocking the beamline. A standard spectral scan was taken to determine the λ max, and a cyclic voltammogram at 50 mv/s was taken to determine the $E_{1/2}$. A cyclic step chronoamperometry experiment was performed by applying 1.2 V vs. NHE and subsequently applying the open circuit potential; the two potentials were cycled up to 10 times as the absorbance at the λ max was collected.

1.4 Inductively Coupled Plasma Mass Spectrometry (ICP-MS)

For post-electrolysis analysis, 1 mL of the electrolyte solution was combined with 2 mL of 70% HNO_3 and heated at 90 °C for 1 hour. The mixture was then diluted with water to achieve a final HNO_3 concentration of 6.7% (v/v). The ruthenium and zirconium contents were measured using an Agilent 7900 inductively coupled plasma mass spectrometer.

1.5 X-ray Photoelectron Spectroscopy (XPS)

XPS spectra were collected on a PHI 5000 Versa probe III spectrometer using an aluminum anode X-ray source with a photon energy of 1486.6 eV. Survey spectra were collected with a 25 W, 15 kV source producing a 100 µm beam with a scan range of 1100 to 0 eV with a step size of 0.5 eV and pass energy of 280 eV. Each elemental range except for the Ru 3p region was scanned for 15 sweeps, whereas the Ru 3p region was scanned for 450 and 900 sweeps for the pre- and post-electrolysis thin films, respectively.

1.6 Scanning Electron Microscopy (SEM)

SEM images were collected with a LEO 1550 field-emission scanning electron microscope (Carl Zeiss, Oberkochen, Germany) at 5.0 kV and a 7.0 mm working distance.

2. Synthesis of Sulfonated Ligand and Catalyst

2.1 3,3'-disulfo-[1,1'-biphenyl]-4,4'-dicarboxylic acid Synthesis

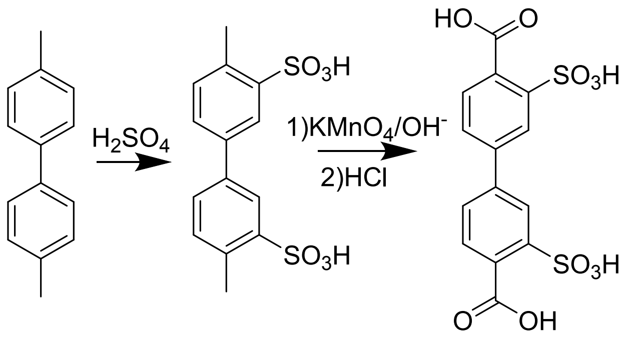

Figure S1: Synthesis scheme of the sulfonated biphenyl dicarboxylic acid through the sulfonation of dimethyl biphenyl followed by oxidation of methyl groups to carboxylic acids.

The sulfonated biphenyl dicarboxylic acid linker, 3,3'-disulfo-[1,1'-biphenyl]-4,4'-dicarboxylic acid, was synthesized via a previously reported procedure.[1] Briefly, 4,4′-dimethyl biphenyl was stirred and heated in concentrated sulfuric acid at 90 °C for 8 h. After cooling to room temperature, 100 mL of acetonitrile and dichloromethane were added to precipitate the product. The solid was filtered and dried then the sulfonated dimethyl-biphenyl was dissolved in an aqueous sodium hydroxide solution before the addition of potassium permanganate. The purple solution was heated to 80 °C overnight and filtered. The filtrate was acidified with HCl and placed in the fridge for 10 hours to precipitate the linker, which was then filtered and washed with 1 M HCl. NMR: (DMSO-d_6, ppm): 7.85 (s,4H), 8.10 (s,2H).

2.2 RuTPY Synthesis

RuTPY, [Ru(tpy)(dcbpy)Cl]PF_6, where tpy = 2,2′:6′,2″-terpyridine and dcbpy = 5,5-dicarboxy-2,2′ - bipyridine, was synthesized following previously reported procedures for Ru polypyridyl water oxidation catalysts used in MOF films and water oxidation studies.[2–4] In the present work, RuTPY was prepared following the same protocol without modification, and no deviations from the reported spectroscopic signatures were observed. Briefly, $RuCl_3 \cdot xH_2O$ was refluxed with 2,2′:6′,2″-terpyridine in ethanol for four hours. The resulting brown precipitate was collected through filtration then dried in a 120 °C oven. The collected Ru(tpy)Cl_3 was dissolved in 3:1 ethanol: water along with 5,5-dicarboxy-2,2′-bipyridine and 1 mL of n-ethylmorpholine. The solution was refluxed overnight, then filtered hot. The filtrate was reduced using rotary evaporation, and 10 mL of 1 M HCl was added to the solution, followed by 10 mL of saturated NH_4PF_6 aqueous solution. The solution was placed in the fridge for 10 hours. The precipitate was filtered and washed with cold 1 M HCl and then dried. ^{1}H NMR: (DMSO-d_6, ppm): 7.31 (m,2H), 7.61 (s,1H), 7.69 (m,2H), 7.99 (t,2H), 8.12 (d,1H), 8.28 (t,1H), 8.69 (m,3H), 8.79 (d,1H), 8.85 (d,2H), 9.15 (d,1H), 10.6 (s,1H). ^{13}C NMR (151 MHz, DMSO-d_6, ppm): 165.68, 164.26, 161.32, 158.38, 157.80, 153.59, 153.01, 151.98, 150.78, 138.95, 137.86, 137.23, 136.03, 135.04, 130.14, 129.64, 128.07, 125.10, 124.46, 123.33. ESI-MS(+) (MeOH): $[M-Cl^-]^+$, m/z = 614.03 (calc. m/z = 614.02).

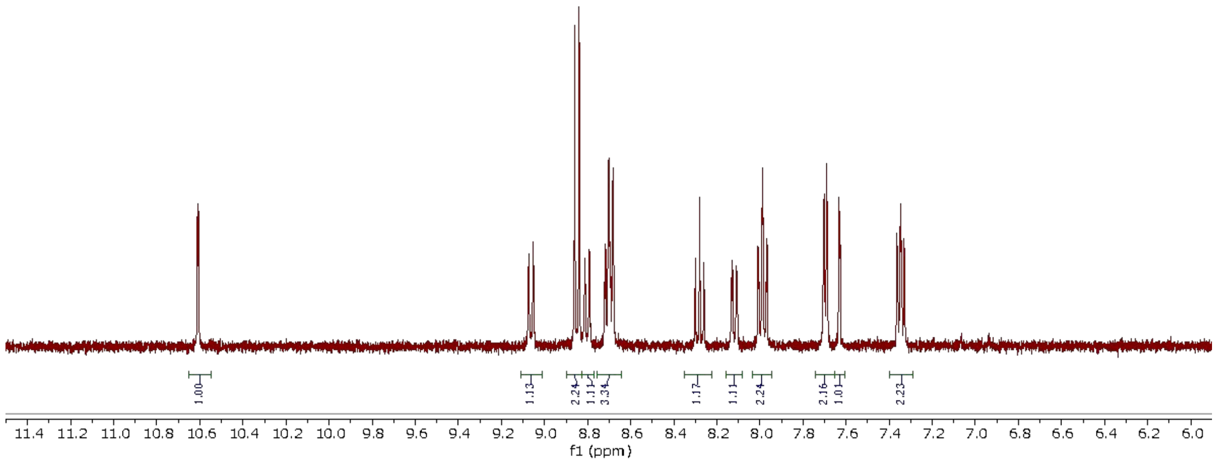

Figure S2: RuTPY NMR.

2.3 RuTPY-UiO-67 Synthesis

RuTPY-UiO-67 was synthesized from a literature procedure.[5] $ZrCl_4$ (21 mg), biphenyl dicarboxylic acid (20 mg), and RuTPY (18 mg) were added to a 6-dram scintillation vial along with 10 mL of dry DMF, and 90 μL of difluoroacetic acid was added, and the solution was sonicated for 10 min. A clean FTO slide was added to the vial, lying diagonally across the solution with the conductive side down. The vial was capped and placed in a 120 °C oven for 24 h. The MOF vial was removed from the oven and allowed to cool to room temperature. The film was removed from solution and washed with DMF and water before being placed in water for 24 h to exchange the Cl ligand for an aqua ligand on the ruthenium catalyst. After that, a vacuum oven was used to dry the film. Finally, the film was stored in water for a full day. The sulfonated MOF, RuTPY-UiO-67-SO_3H was made following the same procedure as the RuTPY-UiO-67 synthesis

with the biphenyl dicarboxylic acid replaced with 3,3'-disulfo-[1,1'-biphenyl]-4,4'-dicarboxylic acid (41 mg).

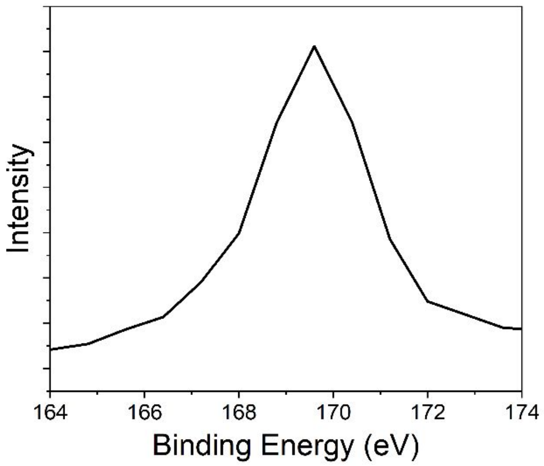

Figure S3: XPS spectrum of RuTPY-UiO-67-SO_3H thin film.

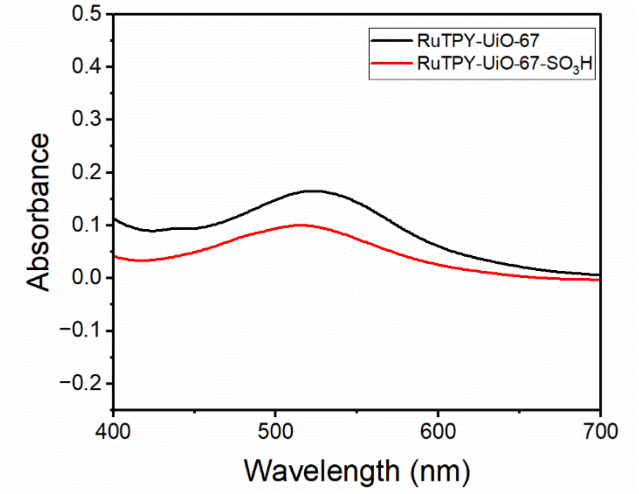

Figure S4: Absorbance spectra of the degraded MOFs.

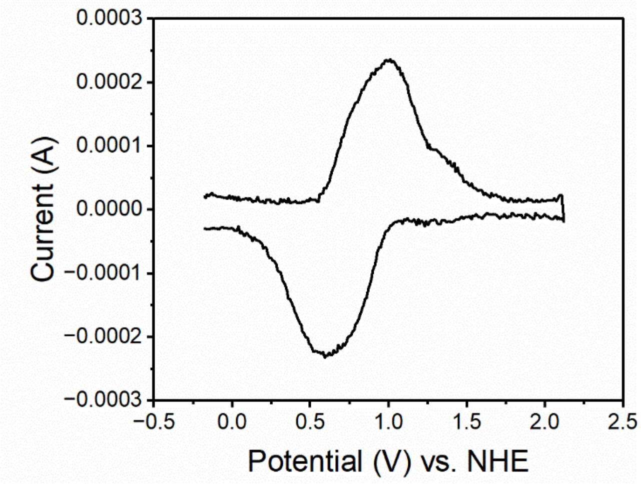

Figure S5: DPV curve of RuTPY-UiO-67-SO_3H.

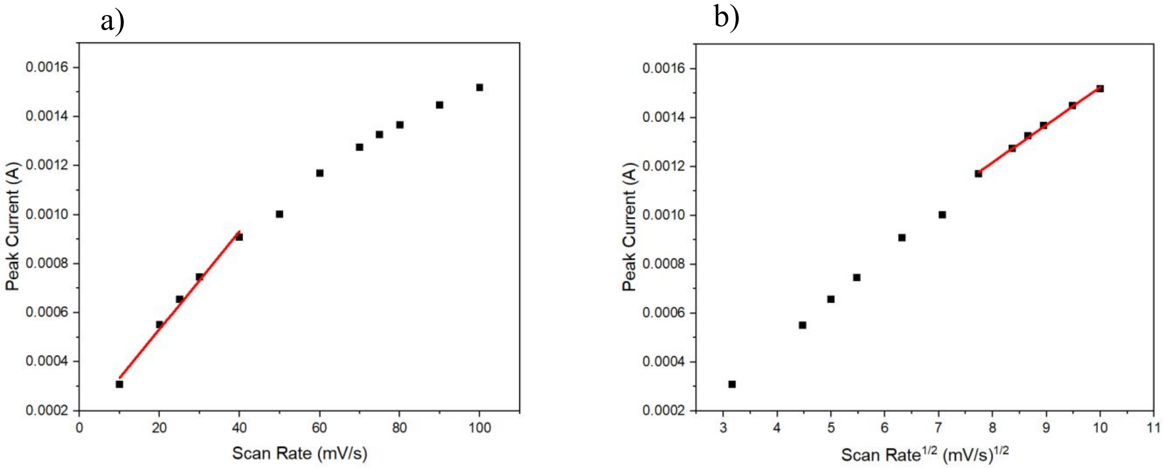

Figure S6: (a) Peak current vs scan rate for RuTPY-UiO-67-SO_3H, (b) peak current vs square root scan rate for RuTPY-UiO-67-SO_3H.

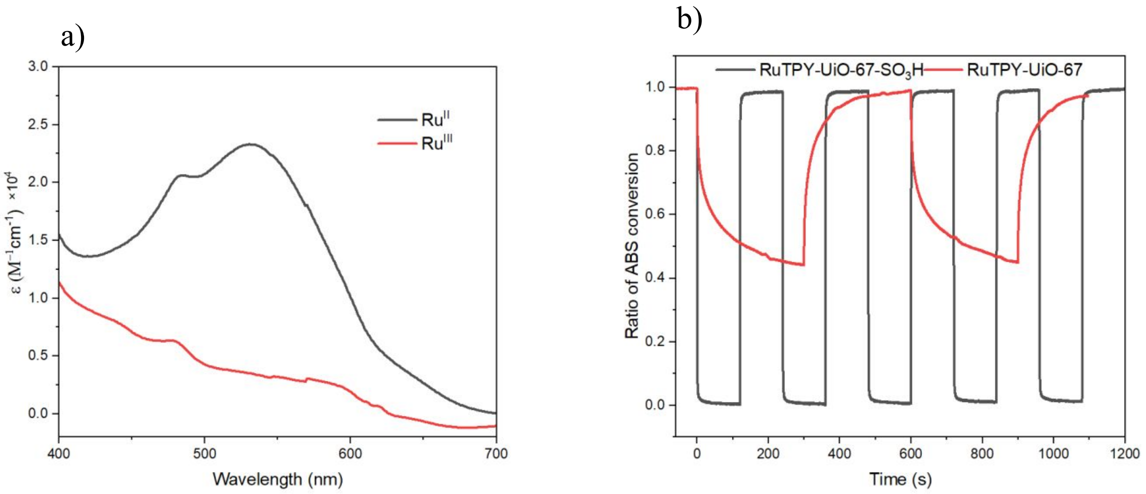

Figure S7: (a) Molar extinction coefficient spectra of RuTPY in the Ru^{II} and Ru^{III} state and (b) Absorbance vs time plot upon oxidation and subsequent reduction.

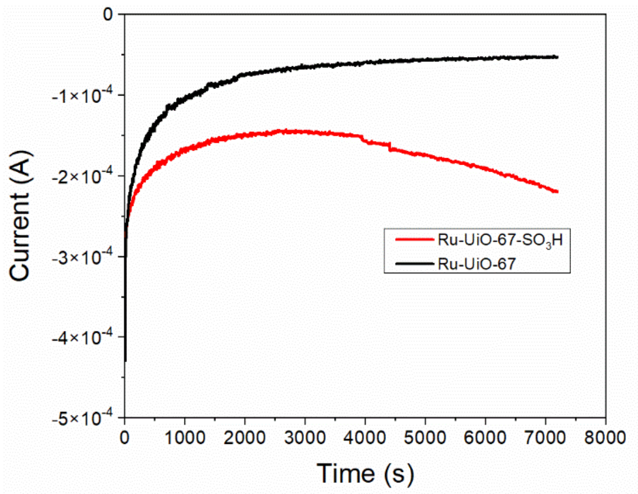

Figure S8: Chronoamperometry plots of bulk electrolysis runs for both MOF films.

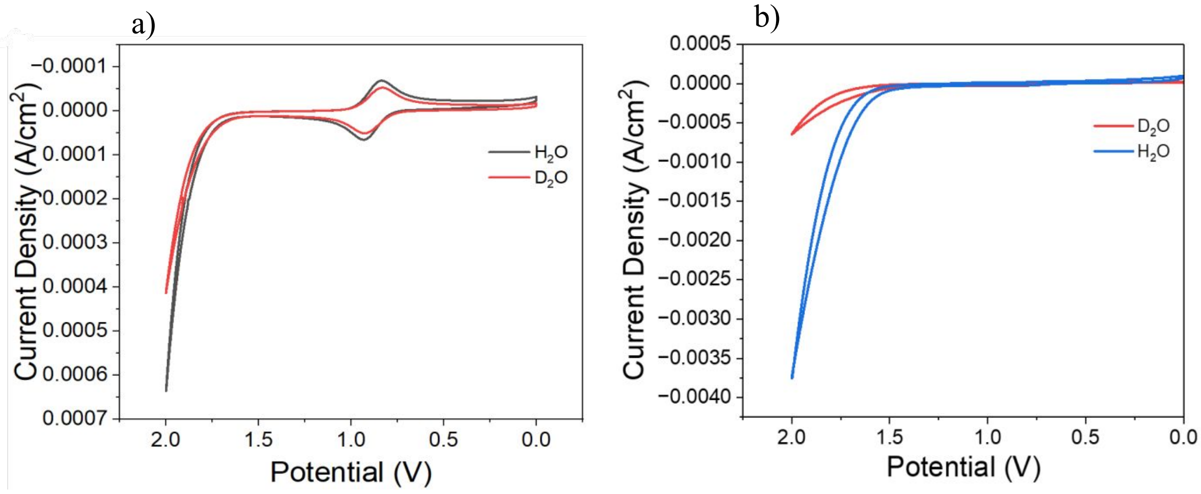

Figure S9: Cyclic voltammograms for (a) RuTPY-UiO-67 and (b) RuTPY-UiO-67-SO_3H thin films in 0.1M $LiClO_4$ in H_2O and D_2O.

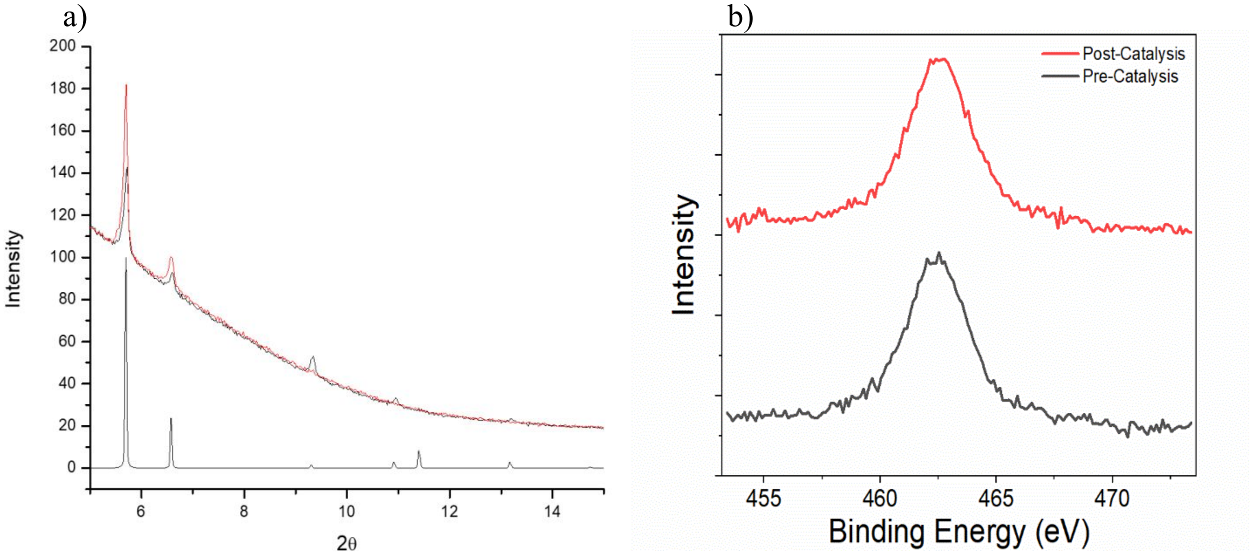

Figure S10: (a) Simulated pattern of UiO-67 (bottom), PXRD pattern of RuTPY-UiO-67-SO_3H film pre-catalysis (black) and post-catalysis (red) and (b) XPS of RuTPY-UiO-67-SO_3H film pre-catalysis (black) and post-catalysis (red) after 2 hours of continuous electrolysis.

References

(1) Zhou, L.-J.; Deng, W.-H.; Wang, Y.-L.; Xu, G.; Yin, S.-G.; Liu, Q.-Y. Lanthanide–Potassium Biphenyl-3,3′-Disulfonyl-4,4′-Dicarboxylate Frameworks: Gas Sorption, Proton Conductivity, and Luminescent Sensing of Metal Ions. *Inorg. Chem.* **2016**, *55* (12), 6271–6277. https://doi.org/10.1021/acs.inorgchem.6b00928.

(2) Gibbons, B.; Cairnie, D. R.; Thomas, B.; Yang, X.; Ilic, S.; Morris, A. J. Photoelectrochemical Water Oxidation by a MOF/Semiconductor Composite. *Chem. Sci.* **2023**, *14* (18), 4672–4680. https://doi.org/10.1039/D2SC06361A.

(3) Johnson, B. A.; Bhunia, A.; Ott, S. Electrocatalytic Water Oxidation by a Molecular Catalyst Incorporated into a Metal–Organic Framework Thin Film. *Dalton Trans.* **2017**, *46* (5), 1382–1388. https://doi.org/10.1039/C6DT03718F.

(4) Lin, S.; Ravari, A. K.; Zhu, J.; Usov, P. M.; Cai, M.; Ahrenholtz, S. R.; Pushkar, Y.; Morris, A. J. Insight into Metal–Organic Framework Reactivity: Chemical Water Oxidation Catalyzed by a $[Ru(Tpy)(Dcbpy)(OH_2)]^{2+}$ -Modified UiO-67. *ChemSusChem* **2018**, *11* (2), 464–471. https://doi.org/10.1002/cssc.201701644.

(5) Thomas, B.; Basak, S.; Smith, Q.; Yan, M.; Morris, A. J. Rapid Redox Hopping Charge Transfer and Electrochromism in a Multivariate Metal–Organic Framework. *J. Am. Chem. Soc.* **2025**, *147* (37), 33655–33665. https://doi.org/10.1021/jacs.5c09275.
